# Measuring and assessing HIV/AIDS stigma and discrimination among migrant workers in Zhejiang, China

**DOI:** 10.1186/s12889-016-3518-7

**Published:** 2016-08-20

**Authors:** Haiyan Xing, Wei Yu, Ya Li

**Affiliations:** 1 Department of Nursing, School of Medicine, Shaoxing University, Shaoxing City, Zhejiang Province China; 2 Institute of Epidemiology, Shaoxing Keqiao District Center for Disease Control and Prevention, Shaoxing City, Zhejiang Province China

**Keywords:** HIV/AIDS, Stigma, Scale, Migrant workers

## Abstract

**Background:**

The aim of this study was to develop a Chinese HIV/AIDS Stigma Scale (C-HSS) and test its reliability and validity among migrant workers in eastern China.

**Methods:**

Nine hundred sixty four migrant workers completed the C-HSS questionnaire in Zhejiang province. The Split-half reliability coefficient (R) and Cronbach’s alpha coefficient (a) for internal consistency of the scale were used. Factor analysis was applied for construct validity. Scores of total and subscales were compared among migrants. Correlation between scores and knowledge of HIV/AIDS was analyzed.

**Results:**

The 24-items scale and the four subscales of C-HSS had good internal consistency (R overall was 0.877, subscales ranged from 0.693 to 0.862; Cronbach’s alpha overall was 0.845, subscales ranged from 0.709 to 0.810). Correlation coefficients between each domain and total score were significant (*p* < 0.01). The cumulative contribution rate was 54.17 % by five public factors based on exploratory factor analysis. Except for the thirteenth item and twentieth item, four public factors were in accordance with the basic conceived concept. The confirmatory factor analysis indicated a good fit to the data for the four-domain structure. Negative correlation existed between the level of HIV/AIDS knowledge and stigma.

**Conclusion:**

The results suggest that the C-HSS is a reliable and valid measure for HIV/AIDS stigma in migrant workers.

**Electronic supplementary material:**

The online version of this article (doi:10.1186/s12889-016-3518-7) contains supplementary material, which is available to authorized users.

## Background

Since 1990s, research on HIV and AIDS-related stigma has increased worldwidely. Many researchers suggest that stigma and discrimination is a barrier to the adoption of precautionary behaviors and reduces the quality of life of people living with HIV and AIDS [1, 2]. Stigma has also been linked to reducing people’s willingness to seek voluntary HIV counselling and testing (VCT) [3].

Migrant workers are prone to have high-risk HIV behaviors, with a large proportion of male migrants visiting commercial sex workers (CSWs) either alone or in a group. Related survey also showed that some migrants including men who have sex with men (MSM), female sex workers, sexual promiscuity, drug addicts and so on are frequently mobile [4, 5]. Migrant workers who had high-risk behaviors spread sexually transmitted diseases or HIV to the general population when they moved from one place to another. Migrants had become an HIV/AIDS transmission’s link dot [6]. Compared with the general population, stigma towards HIV was more serious among migrant population [7, 8].

Reducing HIV-related stigma could potentially increase HIV testing, prevention and treatment [9]. A comprehensive and objective assessment of the stigma situation is the most important prerequisite to carry out anti-stigma intervention measures, eliminate discrimination and control the spread of AIDS [10]. Studies on AIDS discrimination and stigma are changing from using qualitative to quantitative research, with populations involving AIDS patients, students and the general population, although stigma is difficult to define and may be manifested in complex ways [11]. In recent years, many valid measurement tools for HIV stigma and discrimination were researched and developed, most of which are English versions. The scales of Chinese versions take up a very small number [12]. AIDS discrimination among medical college students was measured, however the reliability and validity of the scale were not reported [13]. A scale which was revised from Lau was measured among medical and nursing students [14], and the results showed that the revised scale has good reliability and validity [15]. However the items are too professional to popularize the scale to the general population. Zelaya’s AIDS discrimination scale is a comprehensive scale and is not limited to a single dimension, which was developed from Celentano’s scale (3 domains, 22 items) [11].

Zelaya AIDS discrimination scale included 24 items and four subscales, which had good internal consistency (Cronbach's alpha coefficient was 0.86, the coefficient of each subscales was above 0.7) [16]. Permission was obtained from Dr. Zelaya to translate the HSS from English to a Chinese version. Firstly, two scales of translation were finished independently by one psychology PhD and one social medicine PhD both proficient in English. After their careful discussion, the draft of Chinese version was completed. Secondly, another English expert, possessing rich experience in medical area, translated the draft scale from Chinese to English without knowing the original scale. Finally, the final Chinese HIV/AIDS Stigma Scale (C-HSS) was formed after above three experts’ heated discussion and repeated revise. We used the C-HSS measured 1166 medical college students in a university, the results showed it had good reliability and validity (the split-half reliability of scale was 0.931 and the Cronbach’s alpha coefficient of overall and four subscales was 0.905, 0.869, 0.815, 0.866, 0.794. The test-retest reliability of four subscales was above 0.7 and the scale had good content validity, convergent validity and discriminant validity) [17]. The following study looks at evaluating the reliability and validity of the Chinese HIV/AIDS Stigma Scale (C-HSS) in migrant workers.

## Methods

### Sample and data collection

A cross-sectional population-based study was conducted in Zhejiang province, China in 2013. Using a multistage stratified sampling method, at first, three stratifications were divided by economic level (Gross Domestic Productin) in Zhejiang province. The first stratification: Hangzhou, Ningbo and Wenzhou; the second stratification: Shaoxing, Jinhua, Taizhou and Jiaxing; the third stratification included four other cities. Most rural–urban migrants worked in the first and second stratification cities, so the study was carried out all in the first stratification: Hangzhou, Ningbo and Wenzhou. Jinhua and Shaoxing were sampled randomly among the second stratification cities. In each city a list of work units where migrant workers gathered was drawn up such as shoe factory in Wenzhou, textile industry in Shaoxing. In the company of staff who worked in local Center for Disease Control and Prevention, the researchers investigated migrant workers during coffee break. Questionnaire need to be finished independently within half an hour in the factory meeting room. Interviews were carried out with individuals’ informed consent.

### The questionnaire and scoring

The C-HSS includes 24 questions and four domains: fear of transmission and disease (domain1), association with shame, blame and judgment (domain2), personal support of discriminatory actions or policies (domain3), perceived community support of discriminatory actions or policies (domain4). Response categories for items include ‘strongly disagree, disagree, no opinion, agree and strongly agree’ (5-point Likert scale) and raw scores for items range from one to five, no opinion = 3 [16]. After recording the raw scores, these were transformed in order to provide four scale scores each ranging from one (the lowest stigma) to five (the highest stigma).

The knowledge of HIV/AIDS measured was based on questionnaire from Chinese National Center for Disease Control. The questionnaire consisted of eight questions (correct answer 1 score, a total of eight scores), which was applied in general populations and special populations (such as CSWs, MSM, drug addicts). Some study results related with migrant workers were reported [18–20].

### Statistical analysis

Data were analyzed using SPSS version 18.0 and Mplus version 6.11 software. Reliability and the internal consistency for C-HSS were measured using Cronbach’s alpha coefficient and an alpha equal to or greater than 0.70 was considered satisfactory [21]. Exploratory factor analysis (EFA) and confirmatory factor analysis (CFA) were used to measure the factor structure. EFA was performed using the principal component analysis with varimax rotation. CFA was performed while a four-factor model was specified for the analysis. Goodness-of-fit indicators included: comparative fit index (CFI), Tucker-Lewis index (TLI), root mean square error of approximation (RMSEA), and standardized root mean square residual (SRMR). For the CFI, the recommended cut-off values for acceptable values are >0.90. For the RMSEA, values of <0.05 indicate a close fit and values below 0.11 are an acceptable fit [22]. Pearson’s correlation was performed to identify the relationship between stigma and knowledge of HIV/AIDS, *p* < 0.05 indicate a correlation relationship. Data for this study had been provided as Additional file 1.

## Results

### Participant backgrounds

The 964 migrant workers were approached in the final analysis (response rate 96.4 %). Their mean age and standard deviation was 29.2 ± 9.1 years (18–65 years). The main reasons for rural-to-urban migration were earning money (67.2 %), learning skill (16.3 %) and longing for city life (11.1 %). They came from 25 of the 31 Chinese provinces, including 178 (18.5 %) from Zhejiang, 15.0 % from Jiangxi, 13.4 % from Anhui, 12.3 % from Henan and 10.1 % from Hunan. The demographic characteristics of the study sample are shown in Table 1.

**Table 1:** Demographic characteristics of the study sample

	Number (%)
Age	
18–30 years	593 (61.5)
31+ years	371 (38.5)
Gender	
Male	541 (56.1)
Female	423 (43.9)
Educational status	
Primary	216 (22.5)
Secondary	473 (49.2)
Higher	272 (28.3)
Marital status	
Single	375 (38.9)
Married/cohabiting	553 (57.4)
Divorced/separated/widowed	35 (3.6)

### Scale reliability and validity assessment

Internal consistency was measured using the Split-half reliability coefficient (R) and Cronbach’s alpha coefficient (a). The split-half reliability coefficient for the scale was 0.877, and R for the four subscales ranged from 0.693 to 0.862. Cronbach’s alpha for the subscales ranged from 0.709 to 0.810, and the alpha for the total score was 0.845. Correlation coefficients between each domain and total score ranged from 0.124 to 0.765 (Table 2).

**Table 2:** Internal consistency and correlation coefficient between four domains

	Split-half reliability coefficient (R)	Cronbach’s a	Domain 1	Domain 2	Domain 3	Domain 4
Domain 1	0.862	0.810	1			
Domain 2	0.745	0.776	0.352	1		
Domain 3	0.693	0.709	0.124	0.164	1	
Domain 4	0.765	0.762	0.268	0.486	0.394	1
Total	0.877	0.845	0.663	0.741	0.580	0.765

All correlations were significant at *p* < 0.01

With regard to the validity of the scale, exploratory factor analysis was used to determine the loading and extracted factors from 24 items and the cut-offs for the factor loading was 0.4. Kaiser-Meyer-Olkin measure of sampling showed that the data met the conditions of CFA, which was above 0.7 and *p* < 0.05 (KMO = 0.863, *P* < 0.001). Five factors were extracted based on the principle of Initial Eigenvalues >1. The five-factor structure jointly accounted for 54.17 % of the variance based on exploratory factor analysis. Except for item 13 and item 20, four public factors were in accordance with the basic conceived concept (Table 3).

**Table 3:** HIV/AIDS stigma scale factor structure

Item	Extracted factors					a	a_d_
	Factor 1	Factor 2	Factor 3	Factor 4	Factor 5		
Domain 1: fear of transmission and disease						0.810	
Item 1	0.510						0.804
Item 2	0.791						0.756
Item 3	0.782						0.771
Item 4	0.731						0.770
Item 5	0.739						0.775
Item 6	0.571						0.801
Domain 2: association with shame, blame and judgment						0.776	
Item 7		0.630					0.744
Item 8		0.711					0.731
Item 9		0.750					0.731
Item 10		0.613					0.746
Item 11		0.674					0.732
Item 12		0.522					0.768
Domain 3: personal support of discriminatory actions or policies						0.709	
Item 13				0.323	0.607		0.700
Item 14				0.650			0.645
Item 15				0.796			0.637
Item 16				0.577			0.671
Item 17				0.501			0.668
Item 18				0.602			0.695
Domain 4: perceived community support of discriminatory actions or policies						0.762	
Item 19			0.606				0.747
Item 20			0.072	0.585			0.805
Item 21			0.753				0.698
Item 22			0.750				0.699
Item 23			0.757				0.680
Item 24			0.687				0.716
Eigenvalues	5.559	2.742	2.249	1.420	1.030		
Cumulative %	23.163	34.588	43.958	49.875	54.168		

Factor extraction method: principal component analysis

Rotation method: Varimax with Kaiser Normalization. Cronbach’s Alpha coefficient (a)

Cronbach’s Alpha coefficient if item were deleted (a_d_)

Cumulative contribution ratio (Cumulative %)

The four-factor model, that is domain 1, domain 2, domain 3 and domain 4, was specified and tested by confirmatory factor analysis. The results provided a good fit to the data lending support to the original hypothesized structure of the questionnaire with CFI = 0.915, TLI = 0.901, SRMR = 0.053, RMSEA = 0.042, 90 % CI RMSEA = 0.038 to 0.046.

When we deleted item13 and adjusted item 20 from domain 4 to domain 3 based on the results of EFA (Table 3), the model fit index was CFI = 0.918, TLI = 0.905, SRMR = 0.048, RMSEA = 0.042, 90 % CI RMSEA = 0.038 to 0.046.

### HIV/AIDS stigma

There was no significant difference in the four domains and total scale between male and female migrant workers. However, the scores of domain 2 and domain 3 for the old migrants (over 31 years old) were higher compared to the younger (18–30 years old). The score of domain 1 for single migrants was higher compared to those married or other marital status. The scores of each domain and total scale showed significant difference among workers of different educational status. The overall knowledge of HIV/AIDS scores was 4.48 ± 2.18. There was a negative correlation between the level of HIV/AIDS knowledge and stigma, including the four dimensions of scale and total scale (*p* < 0.05). The mean scores of HIV/AIDS stigma were reported in Table 4.

**Table 4:** The distribution of scores to C-HSS according to different variables (mean ± standard deviation)

	Domain 1	Domain 2	Domain 3	Domain 4	Total
All	3.04 ± 0.70	2.73 ± 0.68	2.69 ± 0.59	2.52 ± 0.61	2.75 ± 0.44
Age					
18–30 years	3.08 ± 0.71	2.68 ± 0.68	2.66 ± 0.59	2.49 ± 0.60	2.73 ± 0.45
31+ years	2.99 ± 0.70	2.81 ± 0.67	2.74 ± 0.59	2.57 ± 0.63	2.78 ± 0.43
*P* value ^a^	0.053	0.005	0.029	0.068	0.100
Gender					
Male	3.03 ± 0.73	2.76 ± 0.69	2.67 ± 0.61	2.55 ± 0.62	2.75 ± 0.45
Female	3.06 ± 0.67	2.69 ± 0.65	2.72 ± 0.57	2.49 ± 0.59	2.74 ± 0.44
*P* value ^a^	0.613	0.073	0.221	0.108	0.532
Educational status					
Primary	3.05 ± 0.68	2.84 ± 0.66	2.76 ± 0.57	2.61 ± 0.64	2.81 ± 0.46
Secondary	3.10 ± 0.67	2.76 ± 0.66	2.70 ± 0.58	2.50 ± 0.58	2.77 ± 0.40
Higher	2.94 ± 0.76	2.58 ± 0.70	2.63 ± 0.63	2.49 ± 0.63	2.66 ± 0.49
*P* value ^b^	0.010	<0.001	0.044	0.044	<0.001
Marital status					
Single	3.14 ± 0.74	2.73 ± 0.70	2.66 ± 0.59	2.53 ± 0.60	2.76 ± 0.44
Married/cohabiting	3.00 ± 0.66	2.74 ± 0.65	2.70 ± 0.59	2.52 ± 0.61	2.74 ± 0.44
Divorced/separated/widowed	2.76 ± 0.83	2.54 ± 0.79	2.89 ± 0.61	2.50 ± 0.68	2.67 ± 0.51
*P* value ^b^	0.001	0.220	0.077	0.936	0.416
Levels of HIV/AIDS knowledge (r) ^c^	−0.157	−0.081	−0.374	−0.236	−0.299
*P* value	<0.001	0.012	<0.001	<0.001	<0.001

^a^ Independent-Samples *T* Test

^b^ One-way ANOVA for equal variance assumed, Kruskal-Wallis test for equal variance not assumed

^c^ Pearson’s Correlation Coefficient (r)

## Discussion

With the rapid development of the economy and promotion of the integration between urban and rural areas, the number of the migrant population has rapidly increased, reaching 239 million and 22.6 million in Zhejiang (ranked 2nd in all provinces) in 2013 [23, 24]. The average age of the migrant population in China was 27.9 years old and the percent of males was 53.1 % [23], which was similar to this study (29.2 years old, male 56.1 %) (Table 1). The frequently moving population results high risk of HIV, such as in Xiamen, where the migrant population accounted for 80.3 % of the discovered HIV-infected persons [25]. According to the experience of some countries in Africa, in the early days, the AIDS epidemic spreads from the areas of high to low incidence. There still exist regional differences in the incidence of AIDS in China. National reporting the number of survival patients infected with HIV accounted for 0.033% of the total population. More than one million survival patients lived in nine provinces (Yunnan, Guangxi, Sichuan, Henan, Xinjiang, Guangdong, Chongqing, Hunan and Guizhou), which accounted for 79.0 % of the total survival patients in China. Seven provinces (Tianjin, Gansu, Inner Mongolia, Hainan, Qinghai, Ningxia and Tibet) reported the number of survival was less than 2000, which accounted for about 1.7 % of the total reports in 2013 [26]. So it is a very important period to prevent the spread of AIDS through the migrant population [27]. Reducing and eliminating HIV-related stigma and discrimination has become a difficult and critical point of AIDS prevention and control.

The translation of the Zelaya’s scale went through a rigorous method and was approved by Dr. Zelaya. Our previous study showed that the Chinese version of Zelaya HIV/AIDS discrimination scale has good reliability and validity among medical students in China [17]. Now the scale was studied in migrant workers, the results showed that the reliability of the overall scale and four subscales was very good, although this study did not provide evidence for test-retest reliability. The hypothesis regarding the item component correlations showed desirable results, as expected the four domains of subscales correlated with the overall scale. The split-half reliability coefficient (R) for the scale was maintained between 0.693 and 0.877, the Cronbach’s alpha coefficient (a) for the scale was maintained between 0.709 and 0.845 (Table 2). When an item was deleted the reliability of the domain decreased (except item20), which confirmed that each item contributed in a positive way to the reliability of the scale (Table 3).

With regard to construct validity, this was found to be satisfactory for both the total score and subscores of the C-HSS. Except for item 13 and 20, the other 22 items attributed to the four domains in consistent with the concept based on CFA (Table 3). The CFA also showed that item 13 (People living with HIV/AIDS in this community should be treated the same by health care professionals as people with other illnesses) [16] was isolated from domain 3, and item 20 (People want to be friends with someone who has HIV/AIDS) [16] was prone to domain 3 rather than domain 4 by factor loading. So we checked out two models, including conceived concept structure (24 items 4 domains) and adjusted structure (23 items 4 domains, deleted item13 and adjusted item 20 from domain 4 to domain 3) based on the CFA. The results both provided a good fit for the data of the questionnaire with the same RMSEA, CFI > 0.9, TLI > 0.9, SRMR < 0.06. According to items 13 and 20, maybe there is a difference between Chinese and Western languages and cultural backgrounds. So cultural adaptation should be carry out before wide use of C-HSS among the general Chinese population.

The mean score for C-HSS was 2.75, higher than the score among medical students (2.07, *t* = 47.22, *p* < 0.001) [17]. The findings indicated that stigma was more serious among migrant workers. Known-groups comparison showed that the total score or subscores of the C-HSS were able to distinguish very well between subgroups of the participants with different ages, educational status and marital status. The study results indicated that older people, those single and people with lower educational status had more serious HIV/AIDS stigma compared to the younger migrants, those married or other marital status and those with better educational status. The findings also revealed there was a negative correlation between the level of HIV/AIDS knowledge and stigma. The average scores of knowledge of HIV/AIDS was 4.48, lower than national standard (6, *t* = 21.54, *p* < 0.001). The lack of knowledge of AIDS will further intensify stigma and fear against AIDS [28]. So an effective way to reduce HIV/AIDS stigma and discrimination was to spread AIDS knowledge, including HIV transmission, non-transmission and prevention knowledge (Table 4). These are consistent with results from other studies carried out in different populations in China [29, 30].

Limitations should be considered when interpreting the results. The sample selected is not random completely because migrant workers are scattered in various work units in China, which is very difficult to comply with random completely sampling principles. So participants were chosen based on the labor-intensive industries where migrants gathered such as shoe factory, textile industry, building industry and services industry. Thence this sampling method might have biased our results. We only surveyed migrant workers in Zhejiang province, so this study population may not be representative of general or other populations in China. Further piloting may be required before widespread use of the C-HSS in low risk populations or the general population.

## Conclusions

This is the first study that describes the psychometric properties of the Chinese version of HIV/AIDS Stigma Scale (C-HSS) among migrant workers in China. The results demonstrated an acceptable reliability and validity and provided a useful tool for monitoring and assessing HIV/AIDS stigma in specific population.

## Additional file

Additional file 1 Data set of the C-HSS (B1-B24) and knowledge of HIV/AIDS (C1-C8). (XLS 447 kb)

## Abbreviations

AIDS: Acquired immune deficiency syndrome
CFA: Confirmatory factor analysis
CFI: Comparative fit index
C-HSS: Chinese HIV/AIDS stigma scale
CSWs: Commercial sex workers
EFA: Exploratory factor analysis
HIV: Human immunodeficiency virus
MSM: Men who have sex with men
RMSEA: Root mean square error of approximation
SRMR: Standardized root mean square residual
TLI: Tucker-Lewis index
VCT: HIV voluntary counseling & testing
